# Cocktail treatment with EGFR-specific and CD133-specific chimeric antigen receptor-modified T cells in a patient with advanced cholangiocarcinoma

**DOI:** 10.1186/s13045-016-0378-7

**Published:** 2017-01-05

**Authors:** Kai-chao Feng, Ye-lei Guo, Yang Liu, Han-ren Dai, Yao Wang, Hai-yan Lv, Jian-hua Huang, Qing-ming Yang, Wei-dong Han

**Affiliations:** 1 Department of Bio-therapeutic, Institute of Basic Medicine, Chinese PLA General Hospital, No. 28 Fuxing Road, Beijing, 100853 China; 2 Department of Immunology, Institute of Basic Medicine, Chinese PLA General Hospital, No. 28 Fuxing Road, Beijing, 100853 China; 3 Department of Geriatric Hematology, Chinese PLA General Hospital, Beijing, China

**Keywords:** CART cocktail immunotherapy, Cholangiocarcinoma, EGFR, CD133

## Abstract

**Background:**

Cholangiocarcinoma (CCA) is one of the most fatal malignant tumors with increasing incidence, mortality, and insensitivity to traditional chemo-radiotherapy and targeted therapy. Chimeric antigen receptor-modified T cell (CART) immunotherapy represents a novel strategy for the management of many malignancies. However, the potential of CART therapy in treating advanced unresectable/metastatic CCA is uncharted so far.

**Case presentation:**

In this case, a 52-year-old female who was diagnosed as advanced unresectable/metastatic CCA and resistant to the following chemotherapy and radiotherapy was treated with CART cocktail immunotherapy, which was composed of successive infusions of CART cells targeting epidermal growth factor receptor (EGFR) and CD133, respectively. The patient finally achieved an 8.5-month partial response (PR) from the CART-EGFR therapy and a 4.5-month-lasting PR from the CART133 treatment. The CART-EGFR cells induced acute infusion-related toxicities such as mild chills, fever, fatigue, vomiting and muscle soreness, and a 9-day duration of delayed lower fever, accompanied by escalation of IL-6 and C reactive protein (CRP), acute increase of glutamic-pyruvic transaminase and glutamic-oxalacetic transaminase, and grade 2 lichen striatus-like skin pathological changes. The CART133 cells induced an intermittent upper abdominal dull pain, chills, fever, and rapidly deteriorative grade 3 systemic subcutaneous hemorrhages and congestive rashes together with serum cytokine release, which needed emergent medical intervention including intravenous methylprednisolone.

**Conclusions:**

This case suggests that CART cocktail immunotherapy may be feasible for the treatment of CCA as well as other solid malignancies; however, the toxicities, especially the epidermal/endothelial damages, require a further investigation.

**Trial registration:**

ClinicalTrials.gov NCT01869166 and NCT02541370.

**Electronic supplementary material:**

The online version of this article (doi:10.1186/s13045-016-0378-7) contains supplementary material, which is available to authorized users.

## Background

Cholangiocarcinoma (CCA) represents a diverse group of highly invasive epithelial cancers arising from different locations within the biliary tree showing markers of cholangiocyte differentiation [1]. Despite CCA is relatively rare, accounting for approximately 3% of all gastrointestinal tumors, the incidence seems to be increasing, especially in the Asian population [2]. Complete surgical resection is the only preferred option for all patients diagnosed with CCA. Unfortunately, most of the patients are not qualified for complete resection because of the delayed diagnosis and advanced stage of the disease. For patients with unresectable or metastatic CCA, combination chemotherapy involving gemcitabine and cisplatin is the current recommended standard care of management, and various targeted agents have also been tested in several phase I and II clinical trials [3, 4]. However, the highly desmoplastic nature of CCA as well as its extensive support by a rich tumor microenvironment and profound genetic heterogeneity contribute to its resistance to chemotherapy and targeted therapy, resulting in poor overall response rate (ORR) and overall survival (OS) [5].

Successful application of chimeric antigen receptor (CAR)-modified T cells in CD19-positive B cell hematological malignancies has demonstrated the potency of this approach for cancer immunotherapy [6–9], and CAR T cells targeting a variety of different hematologic and solid tumor antigens are under active clinical development [10, 11]. Epidermal growth factor receptor (EGFR), a receptor tyrosine kinase playing key roles in the diverse processes that stimulate cell proliferation, differentiation, migration, progression, and survival, is overexpressed in 67–100% of biliary cancers [12], making it a rational target for CART immunotherapy. Hence, we moved forward the trial of CART-EGFR immunotherapy (NCT01869166) in advanced unresectable/metastatic CCA following the safety and feasibility evaluation of CART-EGFR therapy in advanced non-small cell lung cancer [13]. Meanwhile, we raised the question of what the alternative target is if patients with EGFR-positive CCA show resistance or relapse to the CART-EGFR protocol. Besides tumor microenvironment (TME), a very important factor in the regulation of tumor angiogenesis, invasion, and metastasis, cancer stem cell (CSC) is another key factor in CCA that is capable of promoting tumor initiation, self-propagation and differentiation, and resistance to chemotherapy and radiotherapy, which could also be influenced by the interaction of cancer cells, TME, and CSC [14, 15]. CD133 is a member of pentaspan transmembrane glycoproteins first identified in the neuroepithelial stem cells in mice and later in normal human somatic cells and various carcinomas including CCA and serves as a specific molecular biomarker for CSC [16], making it a reasonable target for immunotherapy.

In this manuscript, we report a case in which a patient with advanced unresectable/metastatic CCA achieved an 8.5-month partial response (PR) from the initial CART-EGFR treatment and obtained another 4.5-month PR when switched to the CD133-specific CART immunotherapy (registered as NCT02541370) after the resistance to CART-EGFR therapy was confirmed. Based on this case, we define this EGFR-specific and CD133-specific CART sequential treatment as CART cocktail immunotherapy and recommend a further investigation of its safety and feasibility.

## Case presentation

### Patient and medical history

**Fig. 1:**
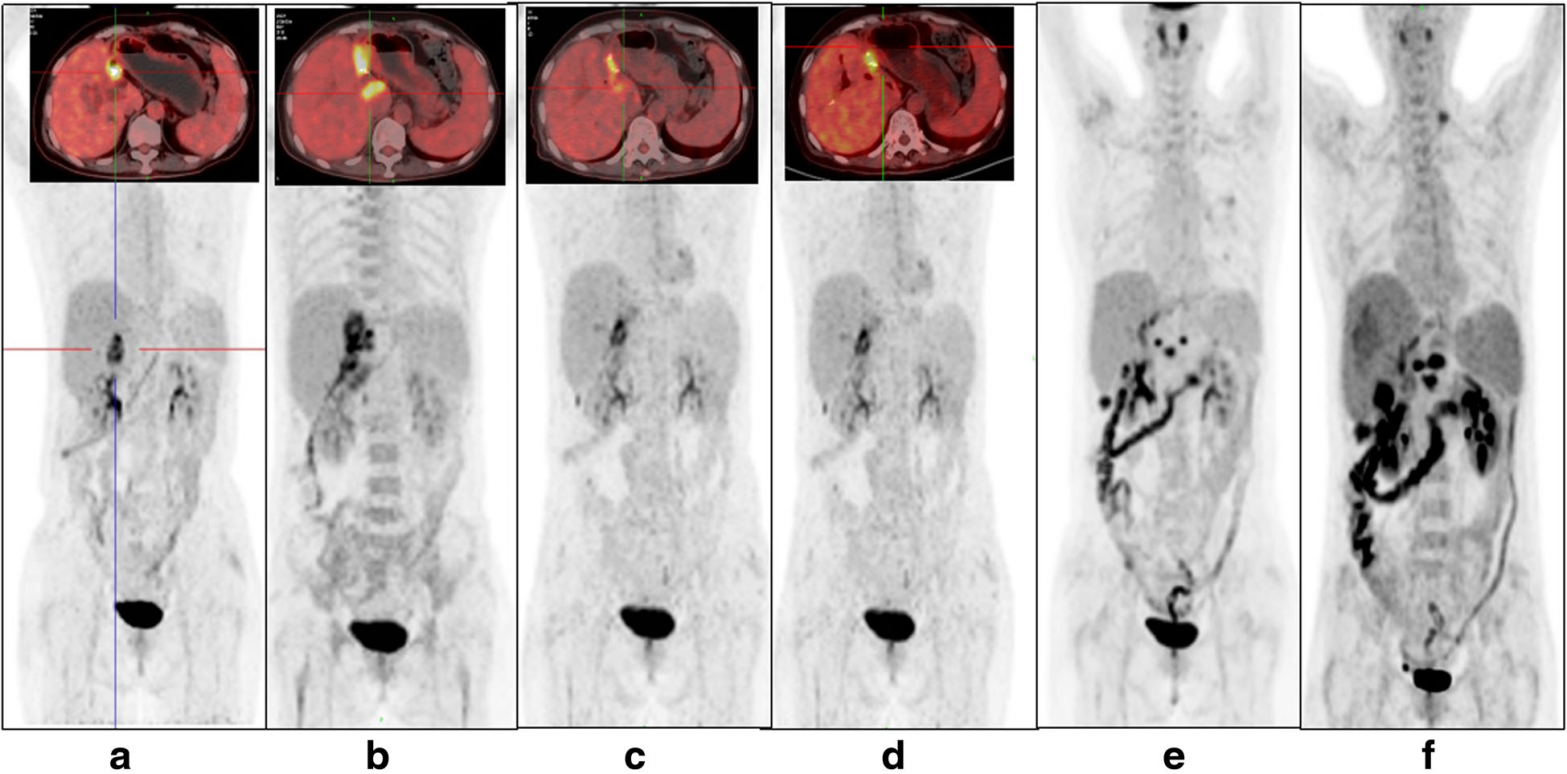
Changes of tumor lesions in sequential PET-CT examinations in the period of CART-EGFR cell infusion. **a** PET-CT examination illustrated hepatic hilar malignancy before surgery. **b** PET-CT examined after the completion of radiotherapy with illustration of a new metastatic hypermetabolic lesion in the hepatic caudate lobe and enlarged soft tissue and retroperitoneal lymph node metastases. **c** PET-CT assessment 6 weeks after the infusion of CART-EGFR cells showed a more than 80% shrinkage of metastatic lesions in the hepatic hilar region and caudate lobe. **d** Routine examination by PET-CT showed a persistent PR status 4 months after the CART-EGFR cell infusion. **e** PET-CT detected multiple new SUV abnormal lesions in the omentum majus, peritoneum, and abdominal cavity 8.5 months after the first cycle of CART-EGFR cell infusion. **f** The examination of PET-CT detected newly emerged metastases in the bottom of the pelvis, right liver lobe, and left supraclavicular lymph node as well as enlargement of previous tumor lesions in the abdomen 4 weeks after the combination of anti-PD-1 antibody and CART-EGFR

A 52-year-old female with history of cholecystectomy and partial resection of the hepatic left lobe in 2004 due to symptomatic gallstone and multiple intrahepatic bile duct cholelithiasis had intermittent fever and progressive jaundice from the beginning of November 2014. Bile duct obstruction and a suspected hepatic hilar malignancy were detected through subsequent ultrasonography, magnetic resonance cholangiopancreatography (MRCP), magnetic resonance imaging (MRI), and positron emission tomography-computed tomography (PET-CT) (Fig. 1a). Exploratory laparotomy was performed at the end of November 2014 (Additional file 1: Figure S1) and found severe intra-abdominal adhesion, a lump (1 × 2 cm) with indurated texture in the proximal left hepatic duct, and disseminated neoplastic lesions infiltrating the hilum of the common hepatic duct, hepatoduodenum ligament, the surrounding lymph nodes, hepatic artery, portal vein, and celiac axis. These findings compelled surgeons to give up radical resection. Purulent bile was discharged from the common duct, and mucus plug protruded from the wall of the bile duct. Partial neoplastic lesions were removed from the enlarged lymph nodes, the nub of the left hepatic duct, and the margin of the common hepatic artery for pathological examination. T duct drainage was installed for relief of biliary obstruction. Pathological reports revealed that the nature of tumor was poorly differentiated adenocarcinoma with differentiation markers of cholangiocyte; in addition, moderate EGFR expression was detected in >90% tumor cells. This patient was finally diagnosed as advanced unresectable perihilar CCA. Five weeks after palliative surgery, the patient received a 4-week course of TOMO therapy on the hilar malignancy (DT = 60 Gy/25 F), one cycle of systematic chemotherapy with albumin-bound paclitaxel alone for her intolerable gastrointestinal toxicities and the infusion of autologous cytokine-induced killer cells. Because of the progressive increase of CA199, PET-CT was re-examined immediately after the completion of radiotherapy and showed a new metastatic hypermetabolic lesion in the hepatic caudate lobe and enlarged soft tissue and retroperitoneal lymph node metastases with abnormal standardized uptake value (SUV) between the liver and stomach when compared with the PET-CT prior to radiotherapy (Fig. 1b). Four weeks following radiotherapy, the patient was enrolled in the CART-EGFR trial. Her performance status was 1 according to the criteria of Eastern Cooperative Oncology Group (ECOG). During the preparation of CART-EGFR cells, she developed high fever, aggressive jaundice, and obstruction of the biliary tract, accompanied by the continuous elevation of CA199 (from 100.5 to 413.5 U/ml), aggressive increase of total bilirubin, direct bilirubin, and other biliary obstruction-related enzymological indexes and was treated with effective broad-spectrum antibiotics and endoscopic biliary stent placement in the hepatic bile ducts.

### Clinical design and protocol eligibility requirements

The trials (ClinicalTrials.gov identifier NCT01869166, NCT02541370) were approved by the Institutional Review Board at the Chinese PLA General Hospital. No commercial sponsor was involved in the studies. The enrolled patients provided written informed consent according to the Declaration of Helsinki.

### Construction of the chimeric antigen receptor and preparation of CART-EGFR and CART133 cells

The DNA sequence of single-chain fragment variable (scFv) targeting EGFR antigen was derived from JQ306330.1 (GenBank Number). Anti-EGFR scFv-CD137-CD3zeta CAR was generated and cloned into the pWPT lentiviral backbone, described in detail by Feng et al. [13]. The construct was verified by DNA sequencing. A pseudotyped, clinical-grade lentiviral vector supernatant was produced by standard transient transfection as McGinley et al. described [17]. According to the manufacturer’s instructions of Lipofectamine 3000 transfection reagent (Invitrogen, USA), pWPT-anti-EGFR CAR plasmid, ps-PAX2 packaging plasmid, and pMD2.G envelope plasmid were transfected into 293 T cells. The lentiviral supernatants were collected and stored at −80 °C. The GFP-CD137-CD3zeta vector was constructed to verify the transduction efficiency by means of FACSCalibur flow cytometry (BD Biosciences). The DNA sequence of scFv targeting CD133 antigen was derived from HW350341.1 (GenBank Number), the generation, construction, and testing of CAR133 T cells followed the same procedure as CART-EGFR cells (Additional file 2: Figure S2).

CART cells were manufactured from autologous peripheral blood mononuclear cells (PBMCs) collected in cell preparation tubes (BD Biosciences, San Jose, CA) purified from 80 to 100 ml whole blood at the Chinese PLA General Hospital Good Manufacturing Practice Facility according to the current standard operating procedures. PBMCs were stimulated with 50 ng/ml anti-CD3 MoAb (Takara, Japan) and 500 U/ml recombinant human interleukin-2 (Peprotech, USA) in GT-T551 medium (Takara). Lentiviral transduction was performed in GT-T551 medium with protamine sulfate (Sigma) for 24 h after 2 days of T cell culture. Afterwards, the cells were further expanded in culture bags (Takara) and harvested as CART cells. Bacteria, fungi, and endotoxin were tested before the infusion of CART cells.

### Flow cytometry

The immunophenotype of CART cells was analyzed by flow cytometry with fluorescently labeled antibodies specific for staining CD3, CD4, CD8, CD45RO, CD62L, and CCR7. Isotype-matched monoclonal antibodies (BD Biosciences) were used for control staining. CAR expression was estimated based on GFP-CD137-CD3zeta-transduced cells in the same batch for all patients by flow cytometry. Data acquisition and analysis were performed using a FACSCalibur flow cytometry (BD Biosciences). In vitro cytotoxicity of CART-EGFR cells was tested by co-culturing with EGFR-positive tumor cells at various effector-to-target ratios in 96-well plates using a 4-h CCK-8 Detection kit (DOJINDO, Japan).

### Quantitative real-time PCR

Quantitative real-time PCR (Q-PCR) was employed to assess the level of CAR fusion gene according to a previously described protocol.13 A 153-bp (base pair) fragment that contains portions of the CD8a chain and adjacent 4-1BB chain (the forward primer 5′-GGTCCTTCTCCTGTCACTGGTT-3′ and reverse primer 5′-TCTTCTTCTTCTGGAAATCGGCAG-3′) was amplified by the ABI PRISM 7900HT Sequence Detection System (Applied Biosystems). Beta-actin was used as an internal control. A 7-point standard curve that consisted of 100 to 108 copies/μl plasmid DNA containing the CAR gene was prepared.

### Cytokine measurements

Serum IL-2, IL-4, IL-6, IL-8, IL-10, IL-12p70, IL-12/IL23p40, IFN-γ, TNF-α, VEGF, and Granzyme B were tested using a BD Biosciences microbead sandwich immunoassay according to the manufacturer’s instructions.

### Results

#### Generation, phenotype, and transfection efficiency of EGFR-specific and CD133-specific CART cells

According to the culturing system as reported previously [13], CART-EGFR cells were generated from the mononuclear cells of 80–100 ml of the patient’s peripheral blood and released for the infusions. Of the infused cells during the first cycle of CART-EGFR therapy, 99.14% were CD3+ cells principally composed of the CD8+ subset (62.26%), and 23.61% were characterized with the central memory phenotype (CD45RO+/CD62L+/CCR7+). In addition, 8.99% of the infused cells were EGFR positive. The phenotype and transfection efficiency of infused CART-EGFR cells in each cycle are documented in Table 1.

**Table 1:** Phenotype and transfection efficiency of the infused CART cells

Cycle	% CAR	CD3^+^ (%)	CD3^+^CD4^+^ (%)	CD3^+^CD8^+^ (%)	CD3^+^CD56^+^ (%)	CD8^+^CD56^+^ (%)	CD45RO^+^ (%)	CD62L^+^ (%)	CCR7^+^ (%)	CD45RO^+^CD62L^+^ CCR7^+^ (%)	CD45RO^+^CD62L^−^ CCR7^−^ (%)
EGFR											
1	8.99	99.14	32.77	62.26	18.98	10.64	77.93	51.01	51.3	23.61	22.42
2	8.98	98.53	16.40	78.29	41.02	36.29	96.99	67.15	71.06	50.82	12.13
3	8.17	98.66	53.66	45.47	7.25	4.26	94.49	76.87	40.96	20.49	3.54
CD133	6.4	95.2	23.6	71.9	11.23	8.19	94.9	74.1	51.4	41.9	12.3

CART133 cells were generated and cultured according to the same procedure of CART-EGFR cells. Of the infused cells, 95.2% were CD3+ cells principally composed of the CD8+ subset (71.9%), and 41.9% were CD45RO+/CD62L+/CCR7+ subset. Besides, 6.4% of the infused cells were CD133 positive. Details are documented in Table 1.

#### The infusion of CART cells and clinical response

**Fig. 2:**
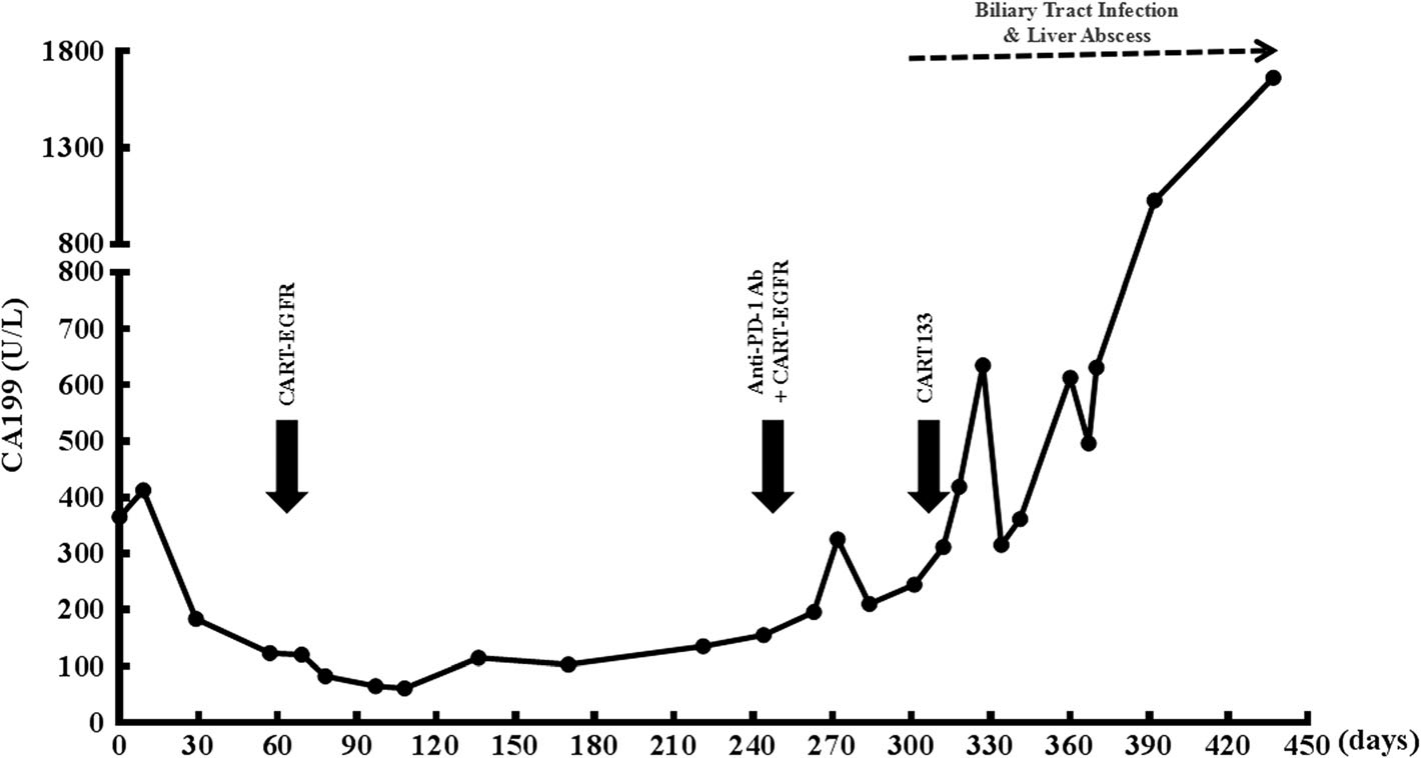
Change of CA199 in the course of CART cocktail immunotherapy

**Fig. 3:**
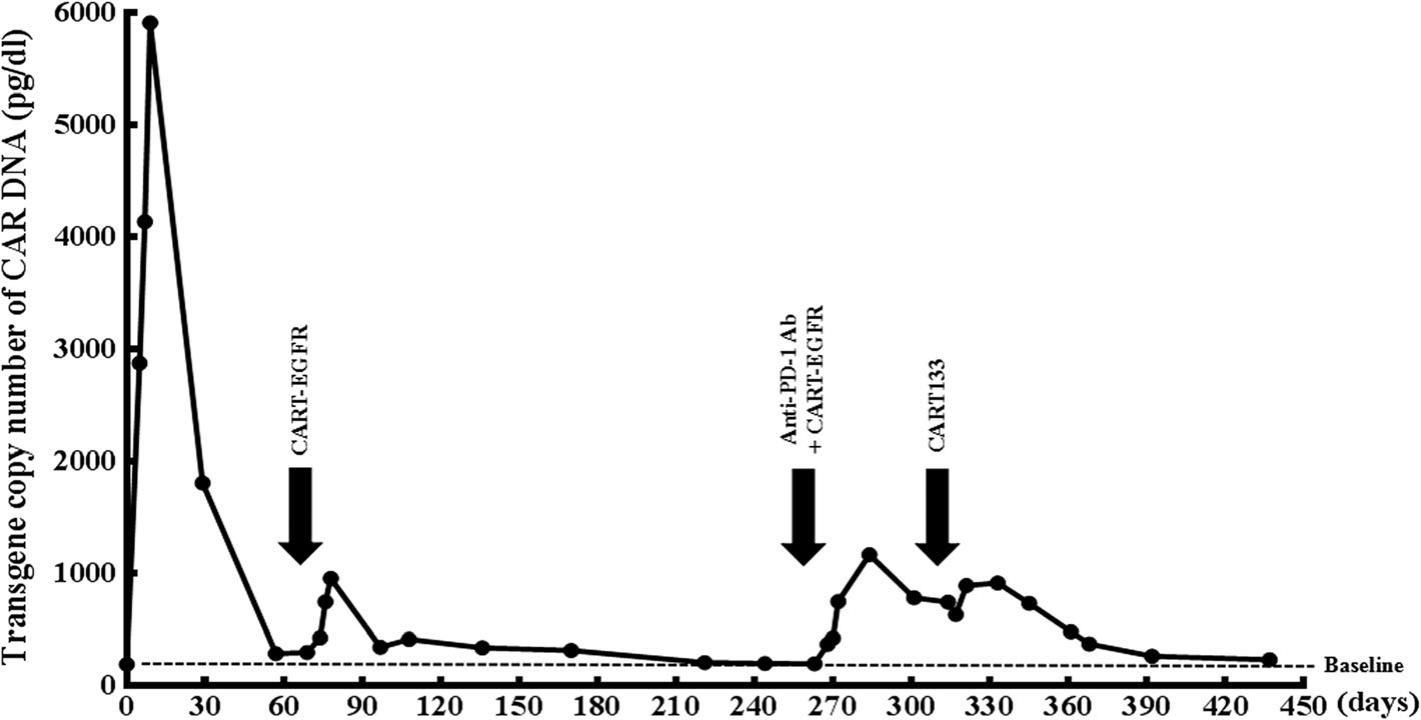
Transgene copy number of CAR DNA in PB throughout the treatment process

**Fig. 4:**
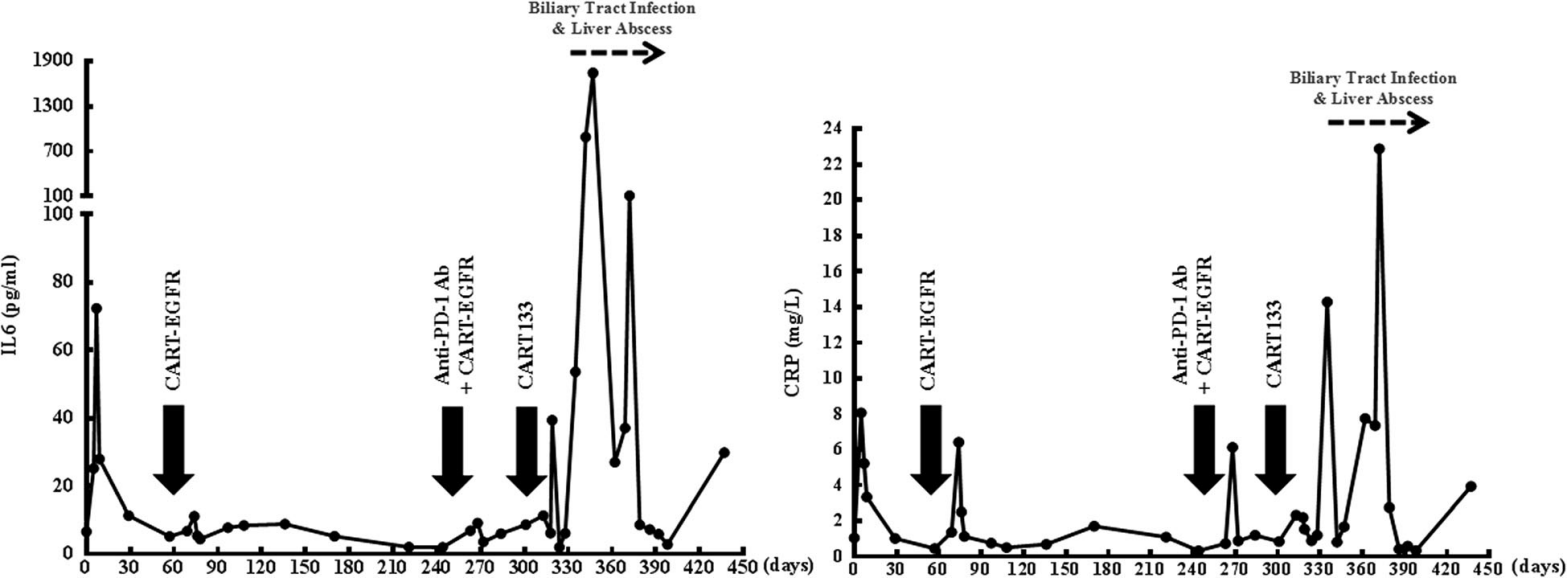
The levels of IL-6 and CRP during and after the infusion of CART-EGFR and CART133 cells

Because of the recently completed radiotherapy within 2 months and intolerance of chemotherapeutic agents, the patient was administered with 4-day successive infusions of 2.2 × 10^6^/kg CART-EGFR cells in total without any conditioning therapy, and achieved a PR in her first assessment 6 weeks later, evaluated according to Response Evaluation Criteria in Solid Tumors version 1.1 (RECIST 1.1) with both MRI and PET-CT, which illustrated a more than 80% shrinkage of metastatic lesions in the hepatic hilar region and caudate lobe and a remarkable decline of the SUV (Fig. 1c), along with a rapid decrease of CA199 from 413.5 to 123.1 U/ml (Fig. 2). The CAR transgene copy number in the patient’s peripheral blood reached a peak of 5916 pg/dl at day 9 accompanied with release of cytokines such as interleukin-6 (IL-6) and C reactive protein (CRP) (Figs. 3 and 4). The second cycle of CART-EGFR monotherapy was administered with a dose of EGFR-CAR+ expressing T cells 2.1 × 10^6^/kg and without any pre-conditioning treatment because the CAR transgene copy number in peripheral blood declined close to the baseline level 2 months after the first infusion of CAR-EGFR expressing genetically modified T cells. Routine examinations of MRI and PET-CT showed a persistent PR accompanied with a further reduction of CA199 to 61.2 U/ml (Figs. 1d and 2). Interestingly, the peak of CAR transgene copy number after the second cycle infusion of CART-EGFR cells did not reach a paralleled or higher level as the first cycle of CART-EGFR therapy did (Fig. 3). The PR status of the patient was maintained for 8.5 months until she developed frequent unmanageable vomiting, upper abdominal dull pain, and gastric acid reflux, which occurred in the middle of November 2015. The subsequent electronic gastroscopy showed duodenal bulb stricture, and PET-CT confirmed tumor progression with illustration of multiple new SUV abnormal lesions in the omentum majus, peritoneum, and abdominal cavity (Fig. 1e).

**Fig. 5:**
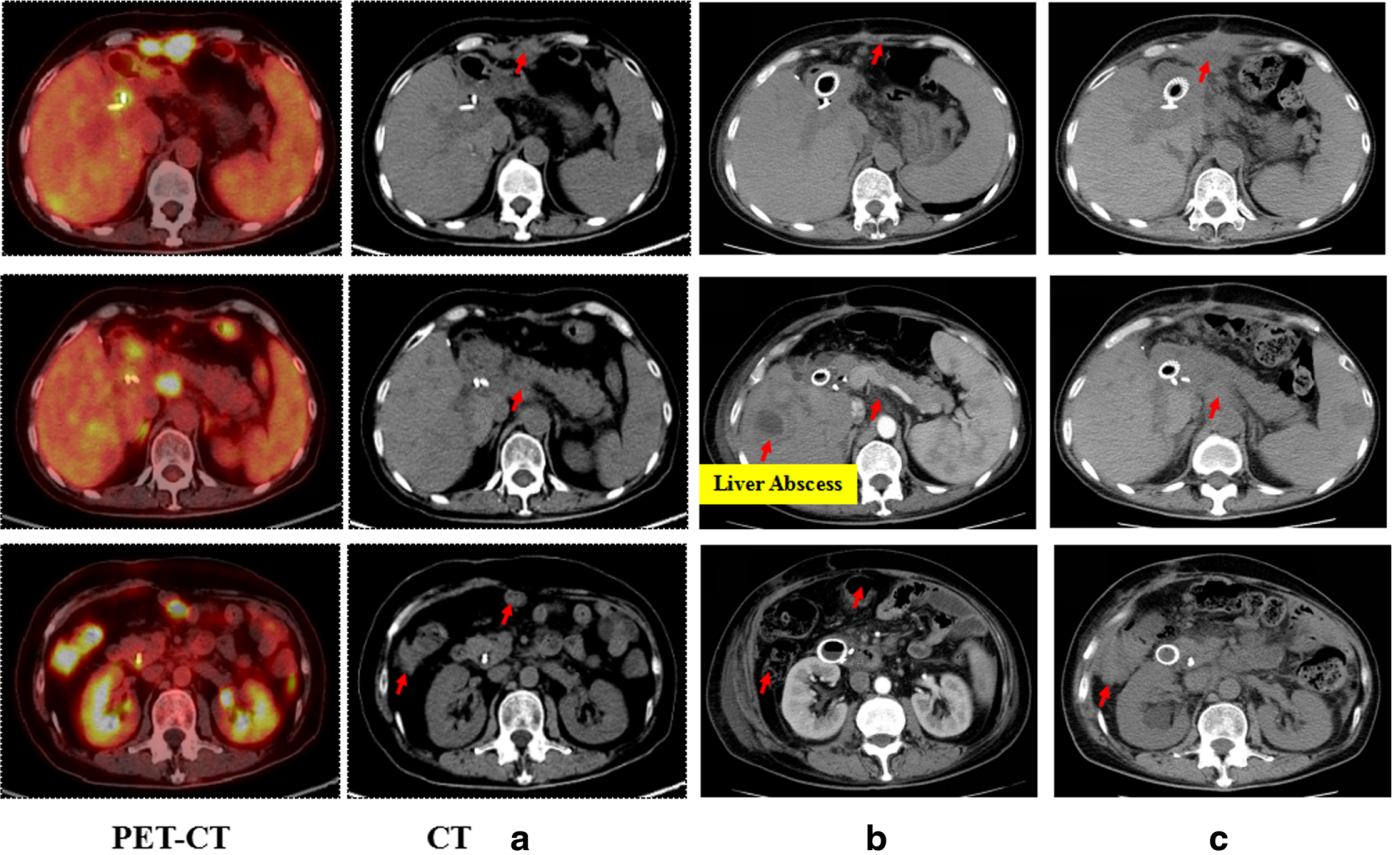
Outcome of CART133 cell infusion. (*a*) With the guidance of PET, tumor lesions were labeled on the images presented by computed tomography (CT) scans before the infusion of CART133 cells (*red arrow*). (*b*) CT images showed remarkable shrinkage or even disappearance of some metastases in the peritoneum and abdominal cavity (*red arrow*) and an abscess in the right liver. (*c*) CT scan detected an approximate 40 × 70 mm new metastatic lesion under the abdominal wall and suspected metastases in the abdominal cavity 4.5 months after the CART 133 immunotherapy (*red arrow*)

Subsequently, the patient was treated with 1 cycle of CART-EGFR therapy combined with 2 cycles of anti-programmed cell death protein 1 (PD-1) monoclonal antibody (Nivolumab, Bristol-Myers Squibb), which was administered at a dose of 100 mg every 2 weeks. Likewise, the CAR transgene copy number monitored in peripheral blood did not reach a similar peak level of the first cycle infusion even combined with anti-PD-1 antibody (Fig. 3). Despite the serum CA199 declined transiently from 326.3 to 210.8 U/ml (Fig. 2), the following PET-CT detected newly emerged metastases in the bottom of the pelvis, right liver lobe, and left supraclavicular lymph node as well as enlargement of previous tumor lesions in the abdomen when compared with the PET-CT before the combined immunotherapy (Figs. 1f and 5a).

Because more than 90% tumor cells expressed CD133 protein (Additional file 3: Figure S3), the patient was then enrolled into the CART133 trial and received the infusion of 1.22 × 10^6^/kg CD133-specific CART cells without conditioning treatment. Because of the occurrence of obstruction in the descending duodenum and the resulting persistent severe biliary tract infection as well as liver abscess, which may interfere with the level of serum CA199 and was treated with antibiotics, the scheduled imaging evaluation was postponed until 2 months after the infusion of CART133 cells, in which contrast-enhanced CT showed remarkable shrinkage or even disappearance of some metastases in the peritoneum and abdominal cavity, leading to an evaluation of PR (Fig. 5b). The CAR transgene copy number in peripheral blood (PB) fluctuated from 377 to 919 pg/dl (Fig. 3). The patient felt persistent dull pain in her upper abdomen 2.5 months later, and the subsequent CT scan detected an approximate 40 × 70 mm new metastatic lesion under the abdominal wall and suspected metastases in the abdominal cavity accompanied by CAR transgene copy number in PB decreasing close to baseline level, drawing a conclusion of progressive disease (PD) (Fig. 5c).

#### Toxicities

##### CART-EGFR

**Fig. 6:**
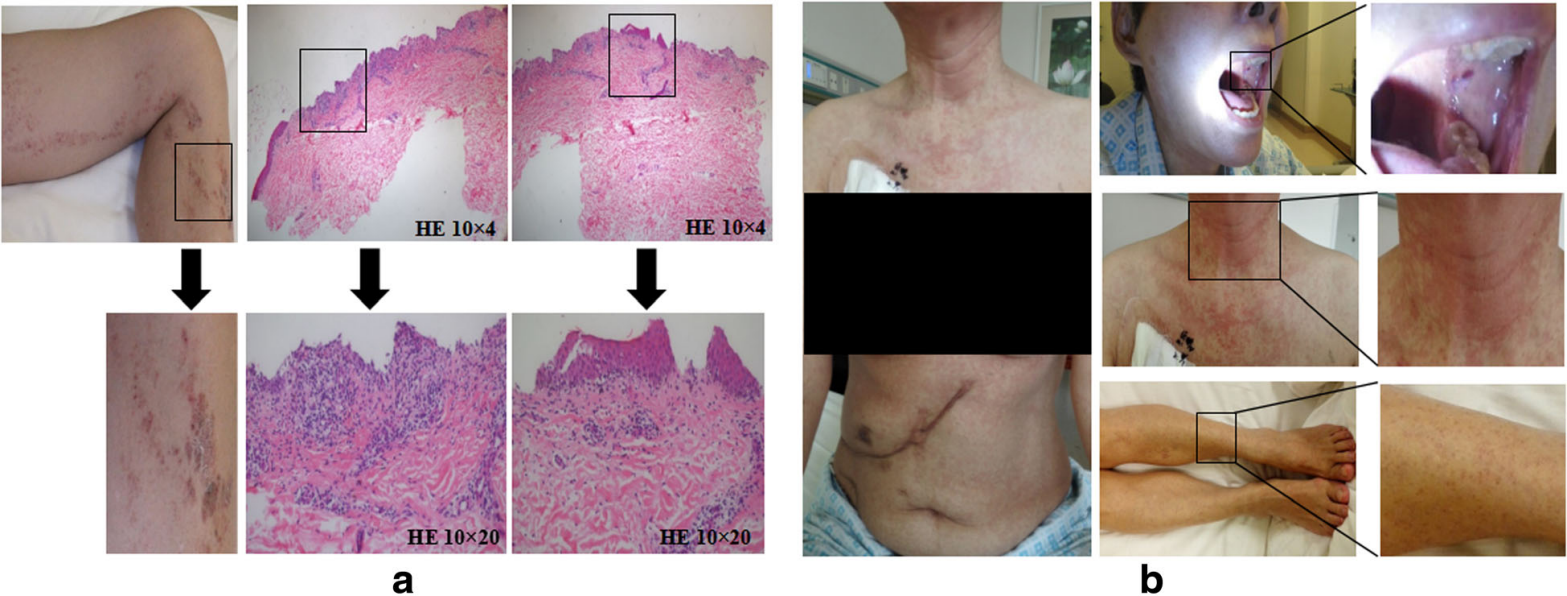
Epidermal and endothelial damages caused by the infusion of CART cells. **a** Lichen striatus-like skin rashes appeared and worsened after the CART-EGFR therapy with the illustration of pathological changes such as the loss of partial epidermis, vacuolar degeneration of basal cells, and infiltration of numerous T lymphocytes in the epidermis and its appendages. **b** Diffused pinpoint hemorrhages and congestive rashes occurred on her neck, right upper arm, chest, left abdomen, and retropharyngeal mucosa after the CART133 cell infusion

Adverse events such as chills, fever, fatigue, vomiting, and muscle soreness occurring during the infusion of CART-EGFR cells were usually mild, tolerable, and manageable by supporting care. However, the patient developed a 9-day lasting lower fever without chills, cough, diarrhea, or any other infectious symptoms since the third day following the CART cell infusion. Repeated laboratory examination of PB showed normal white blood cell count, neutrophil ratio, negative procalcitonin (PCT), and blood cultures, which did not support the diagnosis of infection, but meanwhile escalation of IL-6 and CRP (Fig. 4) and acute increase of glutamic-pyruvic transaminase and glutamic-oxalacetic transaminase (>6ULN). There were a few scattered tiny rashes appeared in the left thigh 2 weeks after the first cycle infusion of CART-EGFR cell, which gradually worsened and became apparent and pruritic with the presentation of multiple flaky or linear rashes spreading from the left thigh to the calf and merging together, which was graded 2 according to the common terminology criteria for adverse events 4.0 (CTCAE 4.0), when the second cycle of CART immunotherapy was fulfilled. The rashes were biopsied with illustration of lichen striatus-like pathological changes such as the loss of partial epidermis, vacuolar degeneration of basal cells, and infiltration of numerous T lymphocytes in the epidermis and its appendages (Fig. 6a), and managed successfully with tacrolimus ointment. No hypotension occurred throughout the CART-EGFR treatment.

##### Combination of CART-EGFR and anti-PD-1 antibody

Except mild nausea, vomiting, chills, fever, and fatigue, there were not any other acute adverse events occurred in the course of the combination treatment. Though dandruff appeared and worsened following the combined immunotherapy, it was not in need of medical interventions. Serum levels of cytokines such as tumor necrosis factor-α (TNF-α), IL-6, and CRP elevated slightly and did not induced any symptoms of cytokine release.

##### CART133

During the infusion of successive dose-escalating CART133 cells over 4 days, there were no any other adverse events except infusion-related mild fever and fatigue. However, on the second morning after the completion of CART133 cell infusion, this patient developed intermittent upper abdominal dull pain, chills, fever (39.1 °C), sporadic pinpoint hemorrhages, and congestive rashes on her calves, which spread rapidly and diffusedly to her neck, right upper arm, chest, left abdomen, and retropharyngeal mucosa accompanied by pruritus and diarrhea in that afternoon and deteriorated furtherly in the following 10 days (Fig. 6b). The skin and subcutaneous adverse events were graded 3 according to CTCAE 4.0. Serial serum cytokine testing detected rapid elevation of TNF-α, IL-6, and CRP (Fig. 4). Etanercept, a fusion protein that acts as a TNF inhibitor, intravenous methylprednisolone, and gamma globulin were administered immediately and successfully reversed the skin/mucosa toxicities.

### Discussion

The use of CAR-modified T cells to eradicate cancers has been studied for more than 20 years until recently CART19 produced promising results in CD19 expressing hematological malignancies. However, how to translate this exciting success of CART therapy to solid tumors remained unclear, requiring a better understanding of potential therapeutic barriers including factors that regulate CART cell expansion, persistence and trafficking in vivo, tumor microenvironment and its interaction with tumor cells, and cancer stem cells as well as conditioning therapies.

As Porter et al. pointed out that vigorous expansion and persistence of CART cells in vivo is a critical determinant of therapeutic efficacy [18], in this case, the CAR transgene copy number in PB ascended to peak level immediately after the beginning of CART-EGFR cell infusion, illustrating that CART-EGFR cells underwent a robust expansion in vivo, which is one of the premises of CART-EGFR cells eliminating EGFR-expressing CCA cells. Besides, the persistence of CART cells in vivo, in spite of a number of possible interference factors such as CAR affinity for the target, CAR immunogenicity, and host-derived factors, has been suggested to be directly correlated with longer time to progression [19]. This patient was treated with the second cycle infusion of CART-EGFR cells as soon as the CAR transgene copy number in PB dropped closely to the baseline level 8 weeks after the first cycle infusion, enabling CART cells effectively to persist in vivo for more than 24 weeks and the patient’s PR status to be sustained for 8.5 months, which may suggest that repeated infusions of CART cells could contribute to prolonging their persistence in vivo and providing a longer progression free survival in conditions where the targeted tumor responded to CART-EGFR cells. Meanwhile, we also found that the CAR transgene copy numbers of the subsequent cycles of CART cell infusion could not reach a parallel or higher peak, even combined with anti-PD-1 antibody, when compared with that of the first cycle, indicating a weakening expansion of CART cells in vivo and loss of CART cells. One potential mechanism is that the murine scFv incorporated in the CAR transgene may lead to the development of CD8^+^ T cell immunity [20].

The ultimate functional competence of CART cells in solid tumors is determined by whether they can effectively penetrate into the tumor microenvironment, a complex and dense fibrotic matrix network orchestrated by both malignant and non-malignant cells, in which the infiltrated CART cells can be inhibited by immunosuppressive cells and molecules such as Tregs, myeloid derived suppressive cells (MDSCs), and programmed cell death protein ligand 1 (PD-L1) [21, 22], resulting in rapid loss of their cytolytic and cytokine secretion capacity and entering a state of hyporesponsiveness [23]. In addition, solid tumors frequently demonstrate metabolic aberrations by overconsuming key amino acids critical for T cell activity and induce hypoxia and a resultant extracellular matrix acidification due to insufficient vascular supply that is hostile to T cell survival [24]. Thus, modulation of tumor microenvironment is necessary to improve outcomes. In this case, despite of the fact that the patient did not receive any direct conditioning treatment with the purpose of transforming the tumor microenvironment, she was treated with 60 Gy radiotherapy prior to immunotherapy with an approximately 1 month interval from the end of radiotherapy to the beginning of CART cell infusion. Although the radiotherapy itself failed to effectively eradicate tumor cells, its direct and delayed effect may play roles in destructing the tumor-supporting stroma, altering the biology of the tumor microenvironment, promoting the release of more tumor-associated antigens, and enhancing immune recognition [25]. Moreover, radiotherapy could make tumors more immunogenic and more susceptible to an improved attack by the immune cells [26], and even generate antitumor effects in those distant metastases which were not locally irradiated (referred to as the abscopal effect) [27, 28]. Hereby, we put forward a hypothesis that radiotherapy could be a reasonable and effective conditioning therapy for improving the outcome of CART therapy.

When the resistance to CART-EGFR therapy was confirmed, we once tried a combination immunotherapy by anti-PD-1 antibody in conjunction with CART-EGFR cell infusion on the basis of a preclinical study that blockade of PD-1 immunosuppression could significantly enhance the therapeutic efficacy of CART cell therapy against established solid tumors [29]. Unfortunately, this treatment ended in failure with PET-CT verifying the progression of disease, although CA199 was transiently decreased. One possible explanation is the unclear level of PD-L1 expression. Recently, PD-L1 expression is highlighted for its value in predicting therapeutic effects of anti-PD-1 drugs. However, the tumor lesion in this case was difficult for biopsy for its anatomic location, resulting that the level of PD-L1 protein expression on tumor cells could not be accurately detected before the initiation of anti-PD-1 therapy, which exist a possibility that PD-L1 expression in tumor milieu might be low level or even negative and therefore lead to the failure of anti-PD-1 treatment. Meanwhile, there were many other parallel checkpoint pathways that could potentially support resistance to anti-PD-1 therapy [30]. Under this situation, selecting a rational target was crucial for the next-step of the patients’ treatment. Recently, the CSCs in CCA have attracted great attention for their highly carcinogenic properties and key roles in mediating self-renewal, tumor re-growth, and therapeutic resistance, consequently, therapy which targeted surface molecular markers and signaling pathways specific to CSC would be a possible potent option for CCA [15, 31]. Among the molecules used individually or in combination to identify cholangiocarcinoma stem cells, CD133 is one of the most important stem cell markers that are associated with higher invasiveness and poorer prognosis [32]. Shien and colleagues also reported that CD133-positive tumor cells showed greater resistance to post-operative treatment than CD133 negative cells, and increased CD133 expression was observed in residual cancer cells after adjuvant therapy [33]. Base on the above, we finally selected CD133 as the target antigen for CART immunotherapy, which in turn produced another partial response, and further demonstrated that CART cocktail immunotherapy may be feasible and rational.

Another crucial issue be of great concern was the toxicity, which could be induced by CART cells on the target antigens expressed on tumor and/or synchronously on normal tissues, termed as on-target off-tumor effect. Damage to normal tissues may even occur by unexpected cross-reaction with a protein that is not expressed on tumor cells [34]. Since the initiation of CART-EGFR cell infusion, this patient experienced not only infusion-related fever, chills, and fatigue, but also sustained pyrexia, acute elevation of serum transaminases, and delayed onset of skin rashes, accompanied by robust elevation of CAR transgene copy number, IL-6, and CRP, although the levels of which did not meet the criteria of diagnosing cytokine-release syndrome [35, 36]. The massive cytokines that might be directly produced by the infused CART cells reflected a strong immune response that could generate both antitumor effects on tumor cells and side effects on normal tissues. In addition, the on-target off-tumor effect caused by CART-EGFR cells was probably the reason for the triggering of dermal toxicities due to the distribution of EGFR on the epidermis [37]. However, all of these adverse events were mild, reversible, and managed with supportive care and topical corticosteroid. The addition of anti-PD-1 antibody did not apparently aggravate the toxicities of CART-EGFR immunotherapy in this case. Nonetheless, in the subsequent treatment of CART133 cell infusion, this patient suffered severe dermatologic, oral mucousal, and gastrointestinal toxicities such as congestive rash and hemorrhage. Although these toxicities were managed successfully by emergent medical interventions including intravenous methylprednisolone and anti-TNF inhibitor, the inherent mechanism may largely be due to the on-target off-tumor effect of CART133 cells which targeted on the CD133 antigen expressed on normal epithelium and vascular endothelium. Meanwhile, it was unclear whether the anti-PD-1 antibody and residual CART-EGFR cells in vivo could play a role in deteriorating the toxicities of CART133 immunotherapy.

## Conclusions

In spite of the mounting achievements in the treatment of hematological malignancies to date, unfortunately, CART immunotherapy is still fraught with many particular obstacles in solid tumors [38], requiring appropriate solutions in order to advance CART cell therapy. The success of this case suggests that CART cocktail immunotherapy may be feasible for the treatment of solid malignancies. However, the known and unknown toxicities, especially the epidermal/endothelial damages, should be further investigated for fully evaluating its safety.

## Additional files

Additional file 1: Figure S1. Diagrammatic sketch of the treatments. (TIF 248 kb)
Additional file 2: Figure S2. Characteristics of CART-133 cells. (A) Schematic representation of anti-CD133 CAR, not to scale. (B) Specific cytotoxicity of CART-133 cells to the CD133-expressing tumor cells. Results of a 4-hour CCK8 analysis at effector/tumor cell (E:T) ratio of 1:1 and 5:1. The effector cells were CART-133, mock, and non-viral transduction T (NT) cells. The target cells were LOVO (CD133−) human colon carcinoma cell line, HepG2 (CD133−) human hepatocellular carcinoma cell line, SW620 (CD133+) human colon carcinoma cell line, and SW1990 (CD133+) human pancreatic cancer cell line. Results are representative as means ± SD (**P* < 0.05, two-way ANOVA test, GraphPad Prism 6.0). (TIF 340 kb)
Additional file 3: Figure S3. CD133 expression tested with immumohistochemical staining. (TIF 1.37 mb)

## Abbreviations

CART: Chimeric antigen receptor-modified T Cell
CCA: Cholangiocarcinoma
CRP: C reactive protein
CSC: Cancer stem cell
CTCAE 4.0: Common terminology criteria for adverse events 4.0
EGFR: Epidermal growth factor receptor
IL-6: Interleukin-6
MDSCs: Myeloid derived suppressive cells
MRCP: Magnetic resonance cholangiopancreatography
MRI: Magnetic resonance imaging
ORR: Overall response rate
OS: Overall survival
PBMCs: Peripheral blood mononuclear cells
PCT: Procalcitonin
PD: Progressive disease
PD-1: Programmed cell death-1
PD-L1: Programmed cell death protein ligand 1.
PET-CT: Positron emission tomography-computed tomography
PR: Partial response
Q-PCR: Quantitative real-time PCR
scFv: Single-chain fragment variable
SUV: Standardized uptake value
TME: Tumor microenvironment
TNF-α: Tumor necrosis factor-α
