# Supplementary figures and images for: Cocktail treatment with EGFR-specific and CD133-specific chimeric antigen receptor-modified T cells in a patient with advanced cholangiocarcinoma
Source: J Hematol Oncol. 2017 Jan 5;10:4. doi: 10.1186/s13045-016-0378-7 (PMC5217546; doi:10.1186/s13045-016-0378-7)

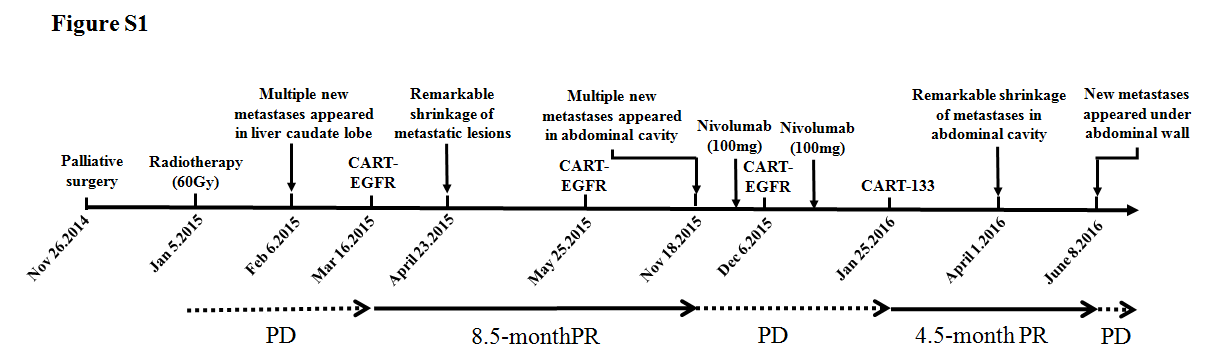

Supplement: Additional file 1: Figure S1. — Diagrammatic sketch of the treatments. (TIF 248 kb) [file 13045_2016_378_MOESM1_ESM.tif]

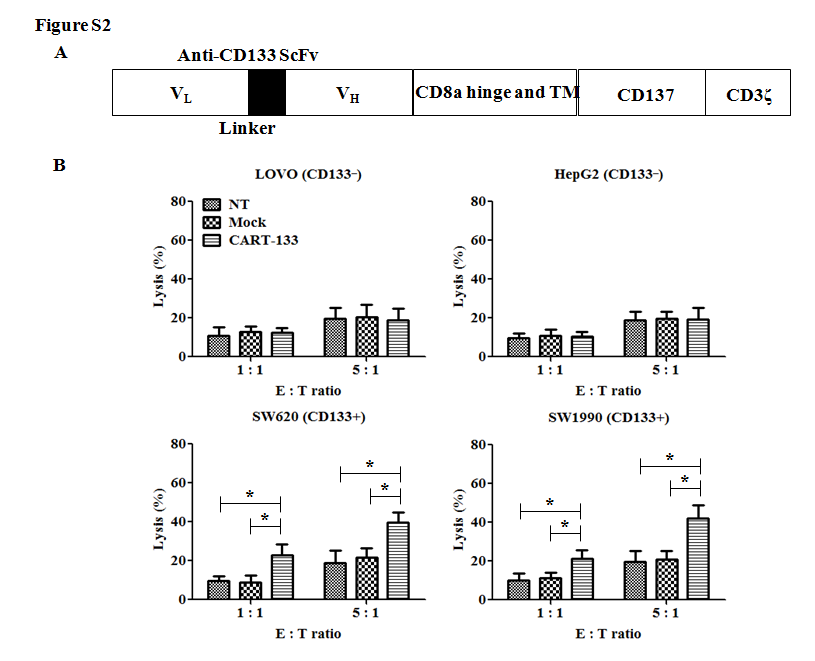

Supplement: Additional file 2: Figure S2. — Characteristics of CART-133 cells. (A) Schematic representation of anti-CD133 CAR, not to scale. (B) Specific cytotoxicity of CART-133 cells to the CD133-expressing tumor cells. Results of a 4-hour CCK8 analysis at effector/tumor cell (E:T) ratio of 1:1 and 5:1. The effector cells were CART-133, mock, and non-viral transduction T (NT) cells. The target cells were LOVO (CD133−) human colon carcinoma cell line, HepG2 (CD133−) human hepatocellular carcinoma cell line, SW620 (CD133+) human colon carcinoma cell line, and SW1990 (CD133+) human pancreatic cancer cell line. Results are representative as means ± SD (*P < 0.05, two-way ANOVA test, GraphPad Prism 6.0). (TIF 340 kb) [file 13045_2016_378_MOESM2_ESM.tif]

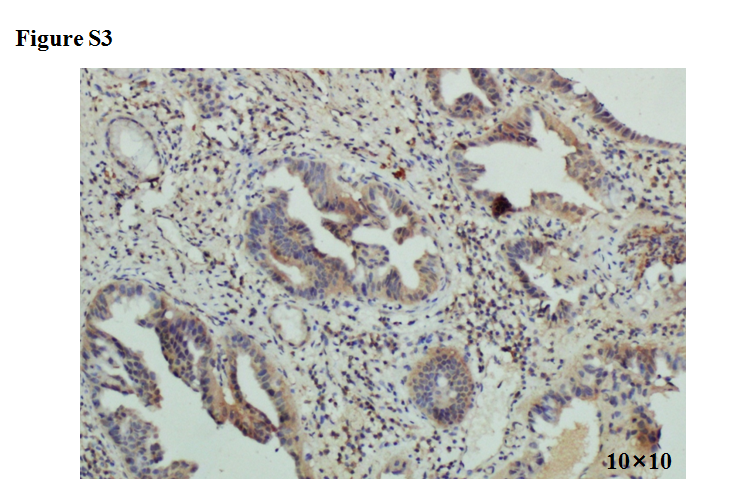

Supplement: Additional file 3: Figure S3. — CD133 expression tested with immumohistochemical staining. (TIF 1.37 mb) [file 13045_2016_378_MOESM3_ESM.tif]
